# Supplementary figures and images for: Blocking Palmitoylation of Toxoplasma gondii Myosin Light Chain 1 Disrupts Glideosome Composition but Has Little Impact on Parasite Motility
Source: mSphere. 2021 May 19;6(3):e00823-20. doi: 10.1128/mSphere.00823-20 (PMC8265671; doi:10.1128/mSphere.00823-20)

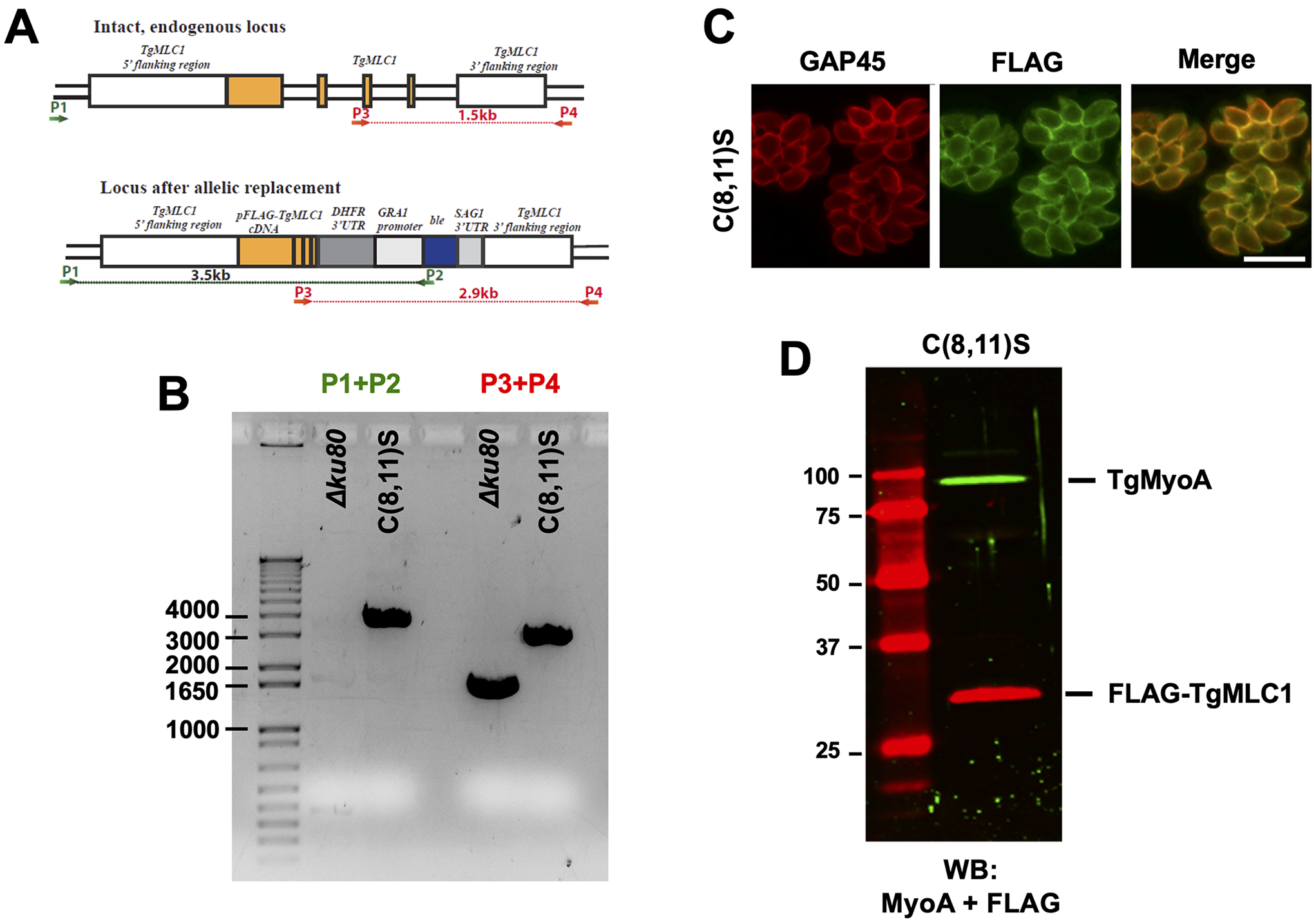

Supplement: FIG S1 [file msphere.00823-20-sf001.tif]

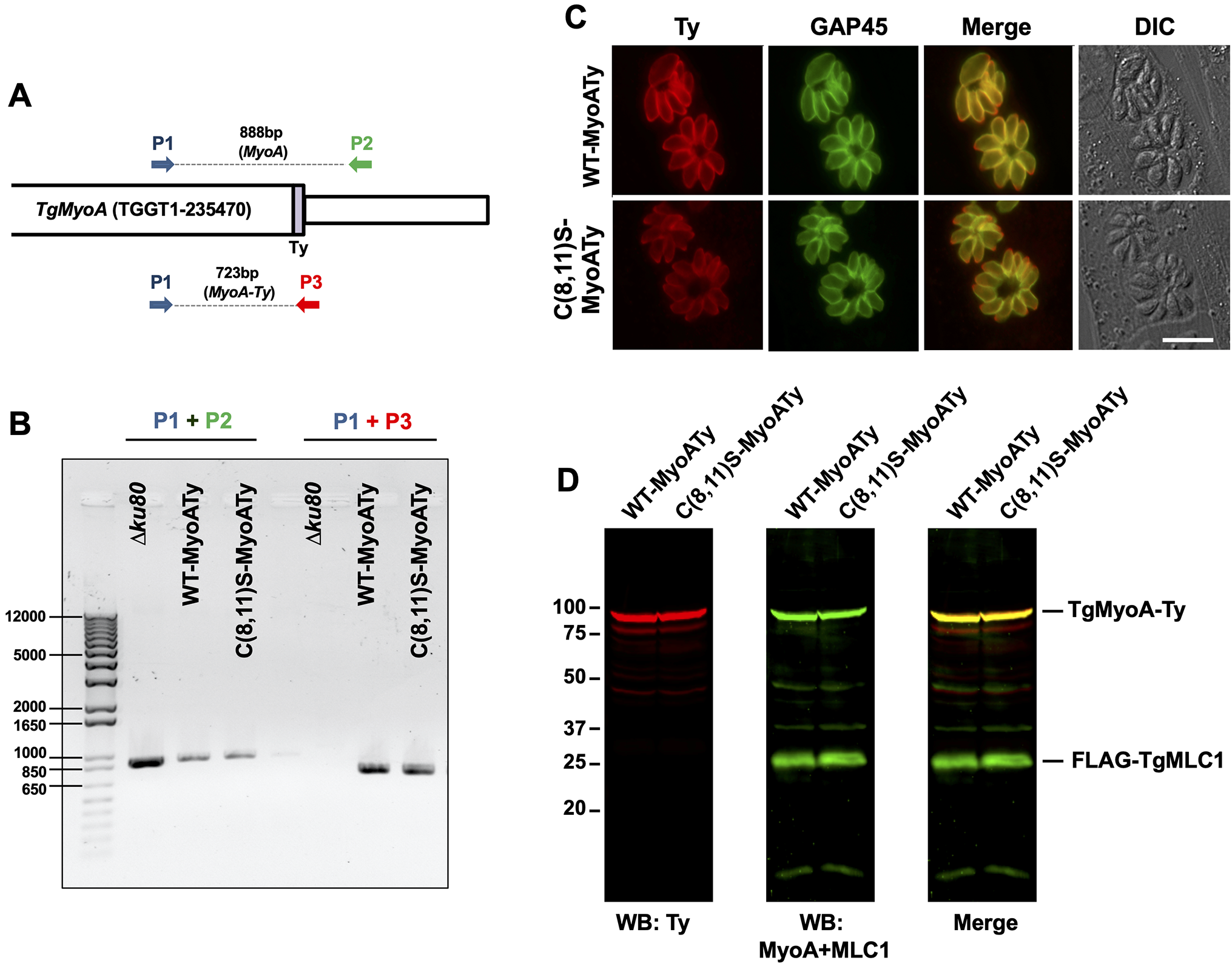

Supplement: FIG S2 [file msphere.00823-20-sf002.tif]

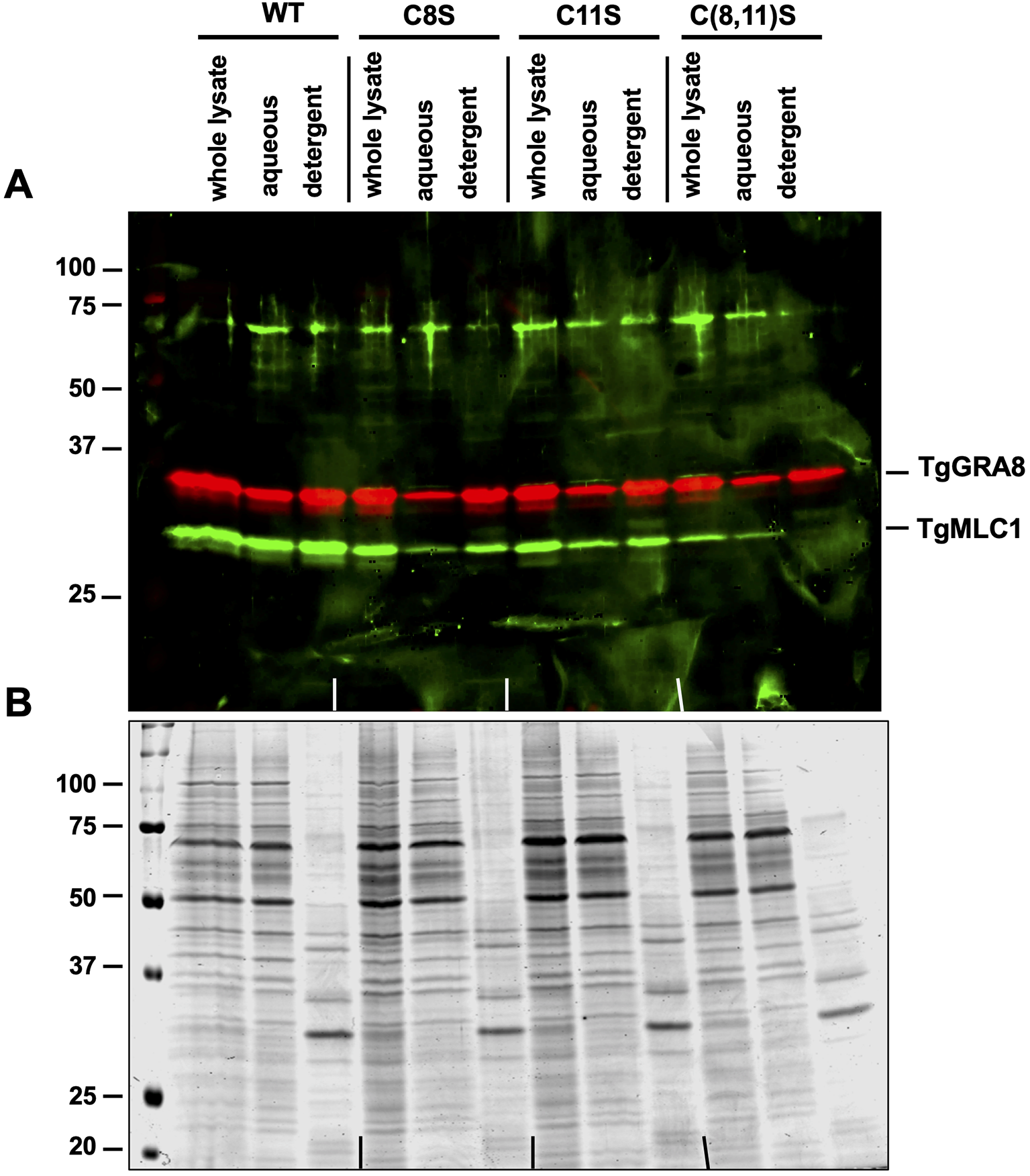

Supplement: FIG S3 [file msphere.00823-20-sf003.tif]

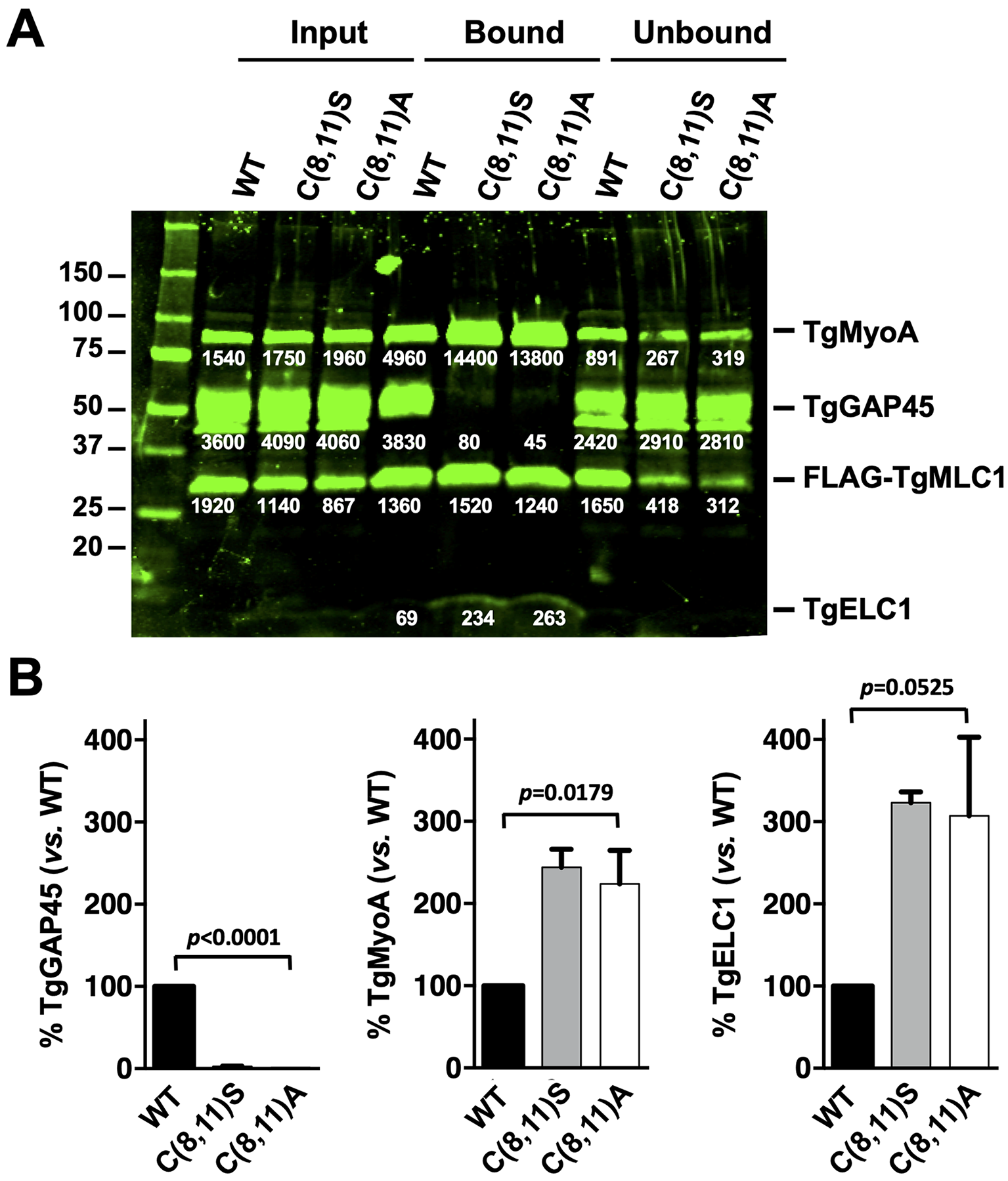

Supplement: FIG S4 [file msphere.00823-20-sf004.tif]

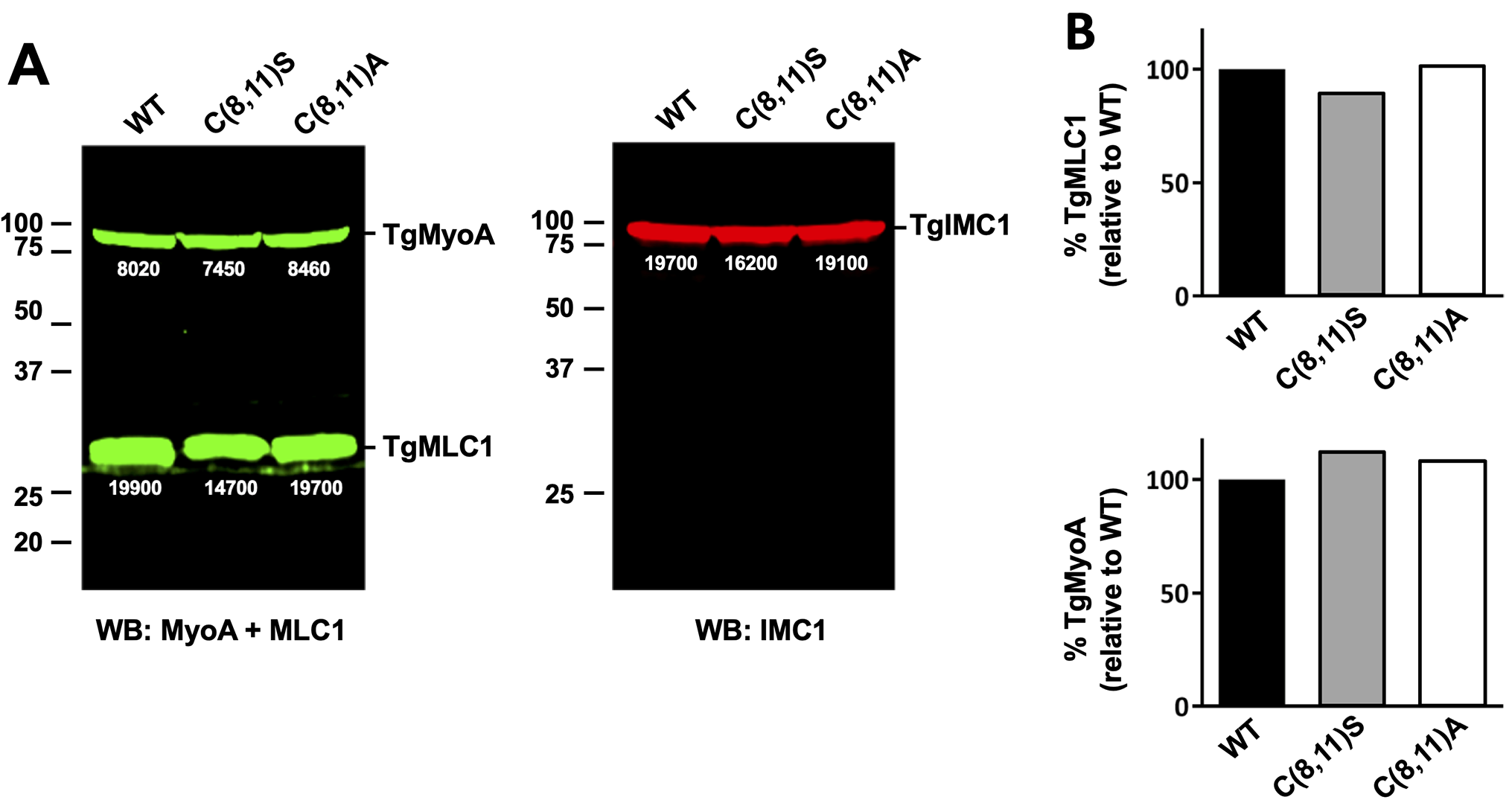

Supplement: FIG S5 [file msphere.00823-20-sf005.tif]

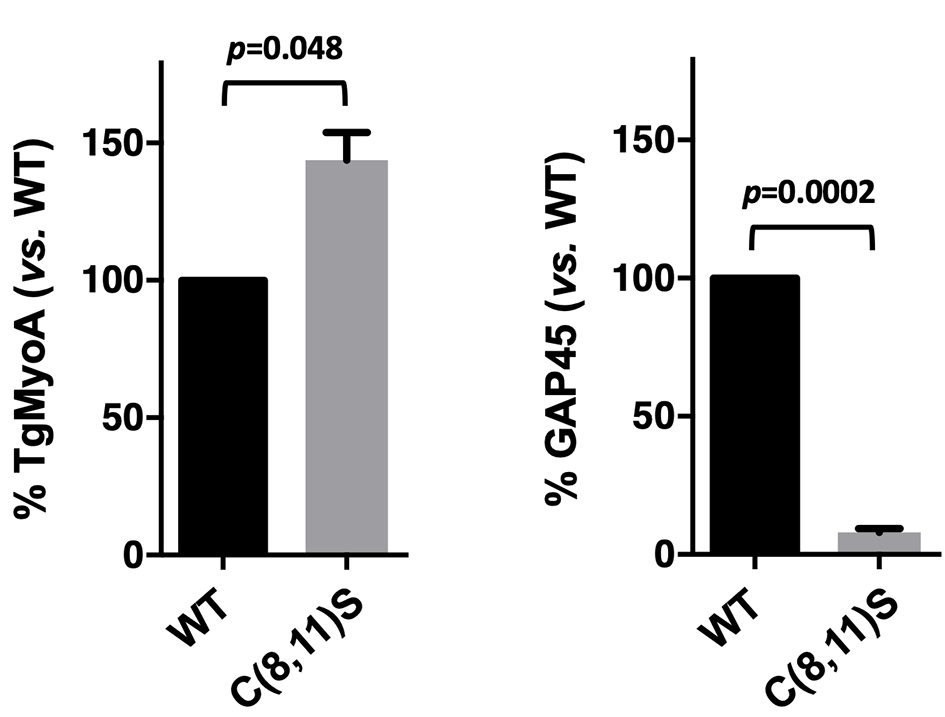

Supplement: FIG S6 [file msphere.00823-20-sf006.tif]

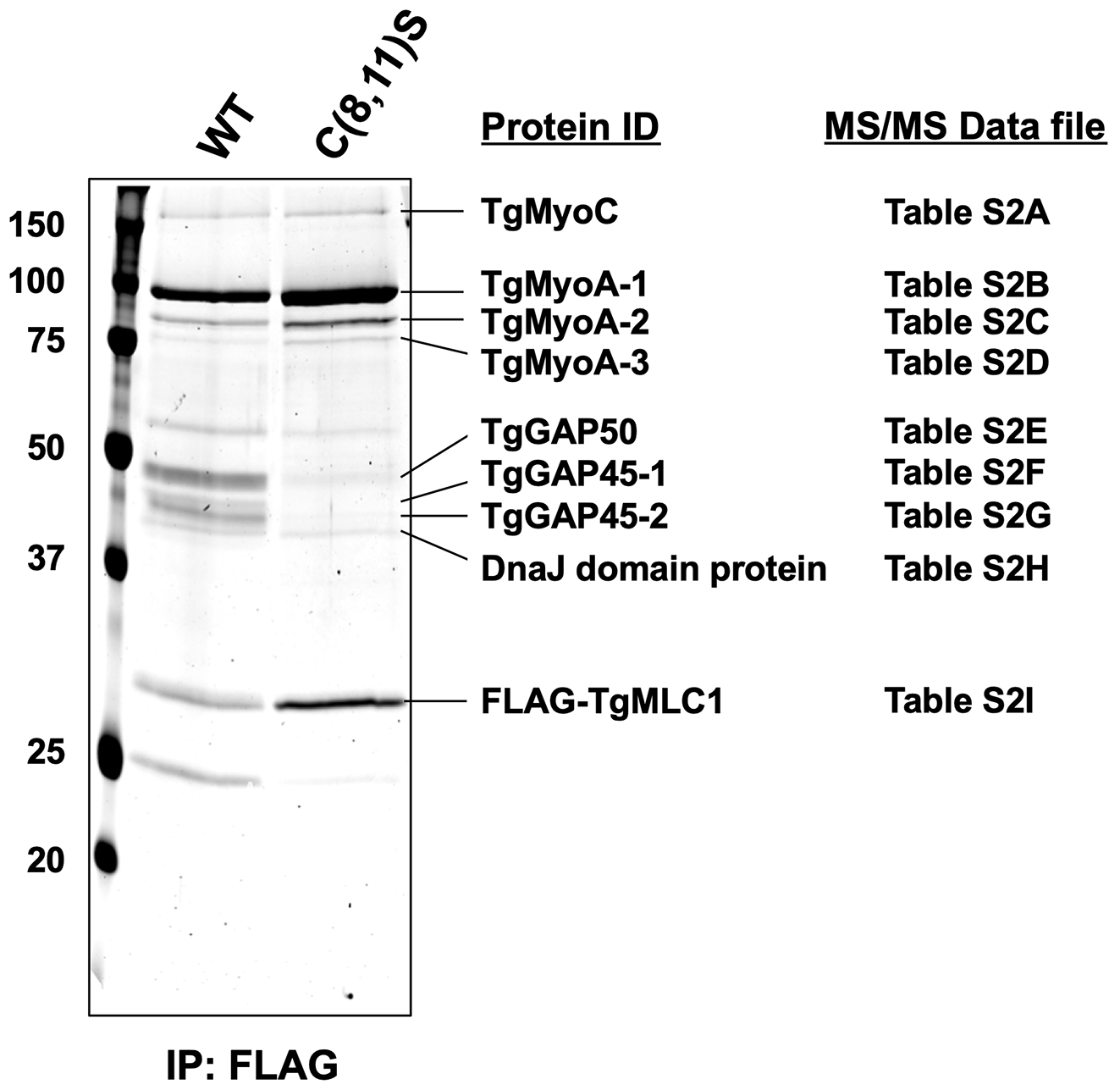

Supplement: FIG S7 [file msphere.00823-20-sf007.tif]

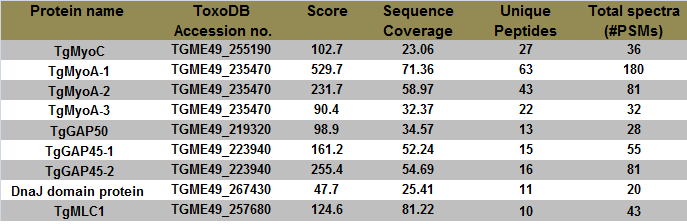


**Supplementary Table 1: Summary of proteins identified in TgMLC1 pulldowns (Fig. S7)**

Supplement: TABLE S1 [file msphere.00823-20-st001.docx]

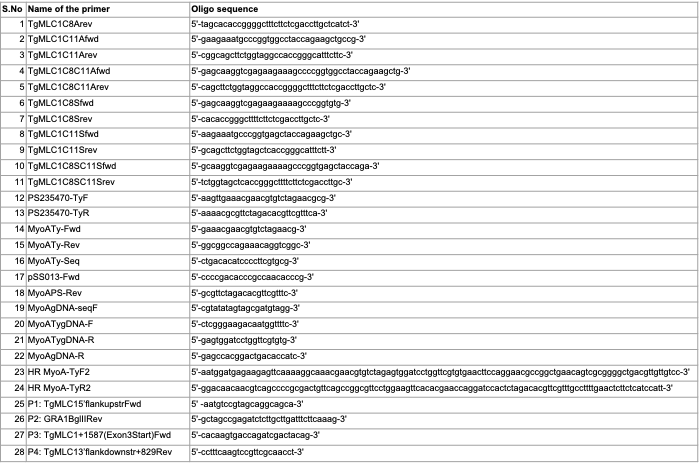


**Supplementary Table 3: Oligonucleotides used in this study**

Supplement: TABLE S3 [file msphere.00823-20-st003.docx]
